# Shiga Toxins and the Pathophysiology of Hemolytic Uremic Syndrome in Humans and Animals

**DOI:** 10.3390/toxins4111261

**Published:** 2012-11-08

**Authors:** Chad L. Mayer, Caitlin S. Leibowitz, Shinichiro Kurosawa, Deborah J. Stearns-Kurosawa

**Affiliations:** Department of Pathology and Laboratory Medicine, Boston University School of Medicine, Boston, MA 02118, USA; Email: cmayer@bu.edu (C.L.M.); csleibow@bu.edu (C.S.L.); kurosawa@bu.edu (S.K.)

**Keywords:** Enterohemorrhagic *E. coli*, Shiga toxins, hemolytic uremic syndrome, animal models

## Abstract

Food-borne diseases are estimated at 76 million illnesses and 5000 deaths every year in the United States with the greatest burden on young children, the elderly and immunocompromised populations. The impact of efficient food distribution systems and a truly global food supply ensures that outbreaks, previously sporadic and contained locally, are far more widespread and emerging pathogens have far more frequent infection opportunities. Enterohemorrhagic *E. coli * is an emerging food- and water-borne pathogen family whose Shiga-like toxins induce painful hemorrhagic colitis with potentially lethal complications of hemolytic uremic syndrome (HUS). The clinical manifestations of Shiga toxin-induced HUS overlap with other related syndromes yet molecular mechanisms differ considerably. As discussed herein, understanding these differences and the novel properties of the toxins is imperative for clinical management decisions, design of appropriate animal models, and choices of adjunctive therapeutics. The emergence of new strains with rapidly aggressive virulence makes clinical and research initiatives in this field a high public health priority.

## 1. Introduction

It has been only thirty years since Shiga toxin-producing *Escherichia coli* was recognized as a human pathogen after the first outbreak investigated as hemorrhagic colitis in 47 patients in Oregon and Michigan [1]. This *E. coli *O157:H7 strain was then considered to be rare, but the 1993 multistate outbreak from undercooked hamburgers at a fast-food chain gained national attention for this newly emerging pathogen [2]. By 2002 there were 350 outbreaks (≥2 cases with same exposure source) reported to the CDC in 49 states, sickening 8598 people with a 17.4% hospitalization rate [3]. Contaminated ground beef and fresh produce account for about 75% of the infections, with the remainder being person-to-person, waterborne or animal contact sources and the vast majority (92%) occurring seasonally from May to November [3]. 

Enterohemorrhagic *E. coli* (EHEC) are not invasive, so bacteremia is rare, but they secrete ribosome inactivating Shiga-like toxins (Stx1 and Stx2 with variants) which are responsible for much of the organ damage [4,5], and Stx2 is more frequently associated with severe disease. Several recent reviews discuss the molecular basis for bacterial success and toxin biochemistry [6,7,8]. Bacterial colonization of the intestine with attaching and effacing lesions support type III secretion of toxins into the vasculature [6]. Micropinocytosis may contribute as toxin antigen is observed in patient gut epithelial cells in the absence of A/E lesions [9]. 

The very young and the elderly are most susceptible to complications and death from EHEC infection. This bacterial infection is the leading cause of acute kidney failure in otherwise healthy children in the US with an average infection rate of 1/100,000. Children less than five years of age are more likely to develop complications requiring hospitalization and kidney dialysis, and the elderly >60 years are more likely to die regardless of the clinical complications [10]. This emerging pathogen has become a global problem as well. The 1996 O157:H7 outbreak in thousands in Sakai City, Japan was purportedly from contaminated bean sprouts distributed in elementary school lunches [11]. The summer 2011 outbreak sourced to bean sprouts in Germany left over 4000 infected [12] and sent a clear warning through the EHEC research and clinical infectious disease communities that more virulent strains are emerging. This O104:H4 strain was a combination of an enteroaggregative *E. coli* strain, normally characterized by intestinal mucosa adherence to cause self-limiting diarrhea [13], that picked up the *stx2 *gene, thereby acquiring high virulence and causing severe clinical consequences, particularly acute kidney injury from hemolytic uremic syndrome and severe neurological abnormalities [14,15]. Karmali *et al.* was the first to make the association between Stx, diarrheal *E. coli *infection and the idiopathic hemolytic uremic syndrome of infancy and childhood [16,17]. This critical link provided the first molecular explanation for the clinical observations, thereby opening new routes for differential diagnosis and pathogen-specific treatment modalities.

Hemolytic uremic syndrome (HUS) is a serious clinical complication of EHEC infection and the severity of a public outbreak is often discussed in terms of the HUS rate. HUS is a clinical composite of thrombocytopenia, hemolytic anemia and thrombotic microangiopathy that contributes to acute kidney injury, often requiring dialysis and can progress to acute renal failure and death. Epidemiology studies have shown that HUS typically develops in about 5%–15% of patients, but this varies between bacterial strains and geographic location. Treatment is supportive, no toxin-specific therapies are available and antibiotics are usually contraindicated, depending on serotype. Recently, a large multi-state prospective study of 259 US children with HUS as a complication of EHEC O157:H7 infection demonstrated unequivocally that exposure to antibiotics of any class in the first week of illness was independently associated with development of HUS and tripled the risk [18]. For the O157:H7 strain, antibiotics increase toxin production due to location of *stx* genes within antibiotic-inducible resident lambdoid prophages [19]. The recent outbreak in Germany with the Stx2-producing O104:H4 strain was notable for its high HUS rate at 22%, occurring overwhelmingly in adults (88%) and most of these being young women [12,15,20]. In this outbreak, use of antibiotics at one hospital center was beneficial, significantly reducing intestinal bacterial colonization duration and improving clinical outcome [21]. Unlike the more common O157:H7 strain, antibiotics reduced Stx secretion from this strain *in vitro* [22]. Argentina has the dubious distinction of having the highest incidence of pediatric diarrhea-associated HUS (13.9/100,000 population) and ~20% of Argentina’s pediatric kidney transplants are the result of EHEC infections [23]. Relative to other infectious disease outbreaks, the death rate from EHEC infection is fairly low at around 3%–5%, but morbidity associated with kidney injury is significant. About 12% of patients with diarrhea-associated HUS progress to end stage renal failure within 4 years and about 25% have long term renal impairment [24,25], putting them at high risk for other clinical complications as eventually encounter pregnancy, high blood pressure, diabetes, cardiovascular disease and other complications of aging. 

Because of the importance of HUS complications, considerable clinical and basic research has focused on this aspect of EHEC infections to define the onset of HUS and to identify cellular dysfunctions that contribute to the pathology. The intent is to identify clinical patterns and molecular biomarkers that identify those at highest risk in order to prevent or mitigate HUS. Not all cases of HUS are due to EHEC infections, and the similarities and distinctions between EHEC-induced HUS and related syndromes of atypical HUS and thrombotic thrombocytopenic purpura can delay diagnosis and complicate clinical management of patients. Identifying effective non-antibiotic adjunctive therapeutics depends on understanding the molecular and pathophysiological mechanisms that contribute to organ injury, and in this respect, development of appropriate and representative animal models is critical. Both of these aspects are discussed below. 

## 2. Distinguishing the Thrombotic Microangiopathy of EHEC-Related HUS

Hemolytic uremic syndrome (HUS) is a clinical syndrome comprising the triad of thrombocytopenia, thrombotic microangiopathy, and hemolytic anemia, and is frequently associated with acute kidney injury. Pathogenic challenge that results in endothelial injury or dysfunction shifts the hemostatic balance toward a pro-thrombotic environment, resulting in thrombotic microangiopathy with inappropriate deposition of clots in microvascular beds causing tissue ischemia. The kidney is a particular target during EHEC infection in part because the toxin receptors are expressed at higher density on glomerular endothelial cells [26] and toxin activities induce endothelial expression of adhesion molecules that support interactions with activated platelets and leukocytes [27] which contribute significantly to clot formation [28]. 

**Figure 1:**
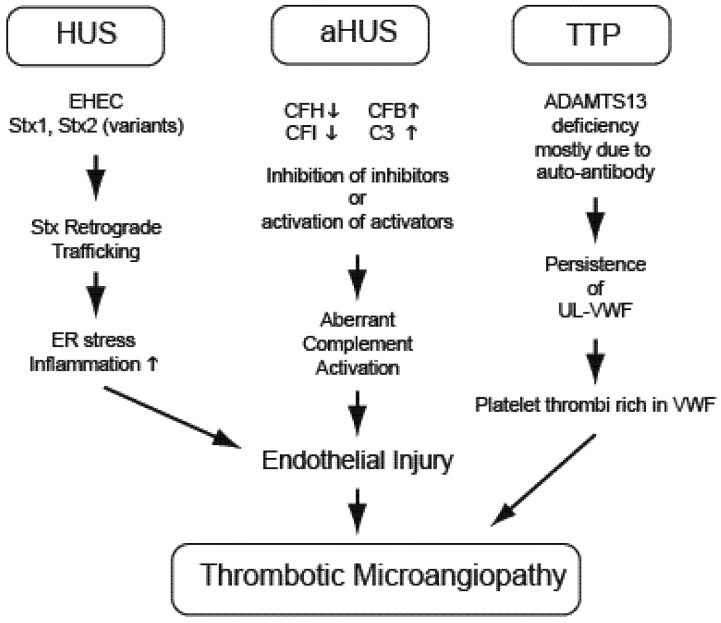
Thrombotic Microangiopathy can result from different molecular pathways. Hemolytic uremic syndrome (HUS), atypical HUS (aHUS) and thrombotic thrombocytopenic purpura (TTP) have shared clinical manifestations, but differing molecular etiologies. EHEC-related HUS initiated by bacterial Stx injures endothelial cells by inducing endoplasmic reticulum (ER) stress responses and transcription events which include generation of inflammatory cytokines and chemokines. Endothelial injury and a pro-thrombotic environment in aHUS results from genetic mutations in complement pathway members and aberrant activation (complement factors H, IB, 3: CFH, CFI, CFB, C3). Coagulopathy during TTP results from inherited or immune-acquired deficiency in a disintegrin and metalloproteinase with a thrombospondin type 1 motif, member 13 (ADAMTS13), needed to cleave vonWillebrand Factor (VWF) released from endothelial cells to prevent accumulation of prothrombotic ultra-large VWF (UL-VWF) oligomers.

Development of a thrombotic microangiopathy can arise as a complication of bacterial infections like EHEC, from genetic mutations in complement regulatory pathways (atypical HUS, aHUS), from deficiencies of regulatory ADAMTS13 enzyme (thrombotic thrombocytopenic purpura, TTP) or even secondarily as a consequence of autoimmune diseases such as systemic lupus erythematosus. These pathways to HUS have different etiologies but frequently overlapping clinical presentation (Figure 1).

**Figure 2:**
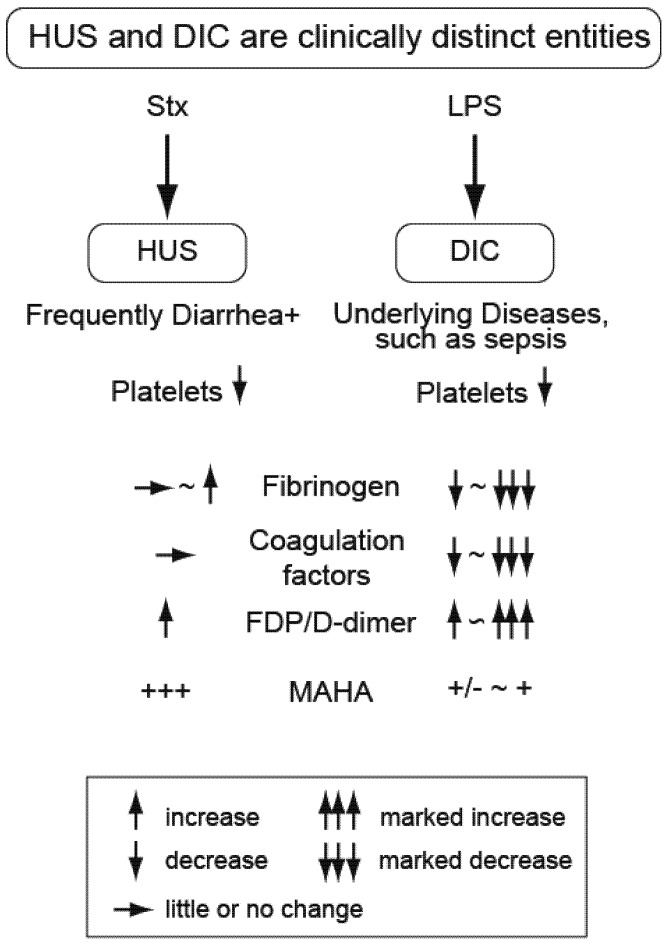
Thrombocytopenia is common to HUS and DIC, but these are clinically distinct entities. Platelet loss occurs with both HUS and DIC, but markers of coagulopathy differ and highlight mechanistic differences. DIC is a consequence of lipopolysaccharide (LPS) in experimental systems (e.g., murine models) or clinically as a complication of underlying diseases such as bacterial sepsis or malignancy. DIC is a consumptive coagulopathy characterized by decreasing fibrinogen and coagulation factors with marked up-regulation of fibrinolysis markers such as fibrin degradation products (FDP) and D-dimer. In contrast, HUS does not consume coagulation factors and fibrinogen levels are often increased in EHEC patients. Moderate levels of fibrinolysis are present, but markers of microangiopathic hemolytic anemia (MAHA), such as schistocytes, are markedly elevated.

Thrombotic microangiopathy is a shared consequence and is related to, but distinct from, disseminated intravascular coagulation (DIC), a consumptive coagulopathy. The coagulopathy of HUS is distinguished from DIC by several parameters, including a normal or elevated fibrinogen level and normal or slightly elevated clotting times because coagulation factors are not consumed (Figure 2). Markers of fibrinolysis (fibrin degradation products, D-dimer) are only modestly increased in HUS relative to DIC, but measures of microangiopathic hemolytic anemia (MAHA) such as schistocytes are markedly increased. Additionally, the renal failure of DIC is attributable to acute tubular necrosis, whereas that associated with HUS is predominately a thrombotic microangiopathy of glomerular vasculature, with or without tubular necrosis. 

Murine models of EHEC toxemia do not develop thrombocytopenia and, therefore, do not present with HUS. Recently these Stx models have included bacterial endotoxin (Gram negative lipopolysaccharide, LPS), tumor necrosis factor-α (TNFα), adenosine diphosphate (ADP) or similar along with the toxin to induce platelet loss in an effort to replicate full-spectrum HUS [29]. While thrombocytopenia is observed in the mice after LPS + Stx, it is a consequence of pro-inflammatory priming, resulting in endotoxin-induced DIC [30], not Stx-induced HUS, with different coagulation mechanisms. 

## 3. Hemolytic Uremic Syndrome (EHEC and Stx)

Over 90% of HUS cases are associated with EHEC infection, also referred to as D + HUS, to indicate that there was diarrhea before the development of HUS. HUS from infection with other pathogens, usually *Streptococcus pneumoniae *(SP-HUS) which exposes the Thomsen-Freidenreich antigen with autoimmune consequences, results in longer hospitalization and long-term kidney injury [31,32]. A diarrheal prodrome can be observed in non-EHEC HUS cases, albeit rarely [33]. Patients usually develop a prodromal hemorrhagic colitis after EHEC contaminated food or water has been ingested [34], with HUS developing after approximately 7–10 days [35]. 

It is widely acknowledged that the Shiga-like toxins produced by the enterohemorrhagic *E. coli* are the main virulence factors driving the organ damage in patients and animal models. EHEC secrete Shiga-like toxin-1 (Stx1) and/or Shiga-like toxin 2 (Stx2), and there are multiple Stx2 variants [36]. The two major toxins are structurally similar with a shared AB_5_ domain structure, but only 56% amino acid homology. Stx are ribosome-inactivating toxins, similar to Shiga toxin from *Shigella dysenteriae* serotype-1 [37] and ricin from castor beans. The enzymatic A subunit and a cell binding B subunit that organizes into pentamers recognize a globotriaosylceramide (Gb_3_) membrane receptor on cells, particularly glomerular endothelial cells [38]. Internalized toxin-receptor complexes undergo retrograde transport to the endoplasmic reticulum via the Golgi apparatus where the A subunit *N*-glycosidase activity removes an adenine from 28S ribosomal RNA to inhibit protein synthesis [39]. A recent study shows Stx1 intracellular trafficking is mediated by Golgi protein GPP130 and disruption of this interaction leads to lysosomal degradation of Stx1 and protection of mice from lethal toxemia [40]. The toxins also induce inflammation in patients [41] and animal models [42], and endoplasmic reticulum stress-induced transcriptional events are stimulated in susceptible cells [43,44]. 

**Figure 3:**
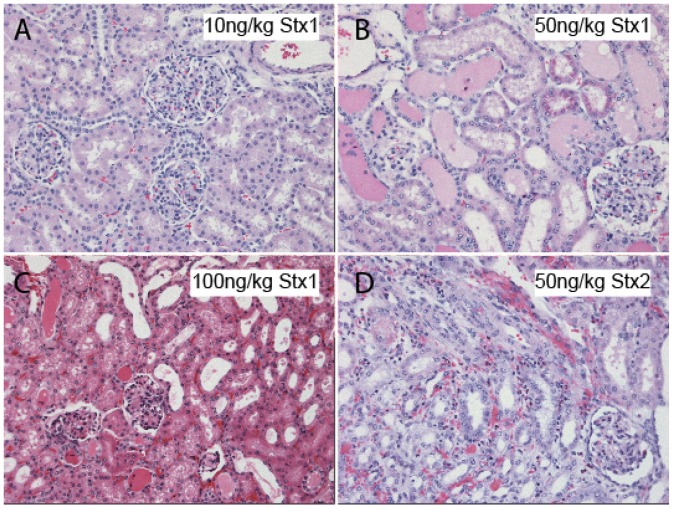
Histopathology of renal injury in nonhuman primates after challenge with Stx1 or Stx2. Light microscopy evaluation of kidney sections from baboons challenged i.v. with different doses of Stx1 or Stx2 reveal dose-dependent kidney injury. (**A**–**C**) Comparison of Stx1 challenges reveals deteriorating glomerular structure, increasing tubular edema and increasing hemorrhage with increasing toxin dose. Clinically, the 10 ng/kg Stx1 dose induced mild and transient effects in the animals, with unremarkable pathology at the light microscopy level. In contrast, a 100 ng/kg Stx1 challenge was lethal within 3–4 days accompanied by fulminant HUS, acute renal failure and systemic inflammatory responses; (**D**) The 50ng/kg Stx2 dose is comparatively severe and 100% lethal by day 5 post-challenge, also with HUS and renal failure. Both toxins elicited leukocyte infiltration and interstitial hemorrhage. Hematoxylin and eosin staining of paraffin embedded tissue sections; 40× magnification.

The Shiga toxins target Gb_3_-rich tubular epithelium, as well as the glomerular endothelial cells, with cellular apoptosis, necrosis and thrombotic microangiopathy contributing to the acute kidney failure seen in patients [45]. In patients, the renal pathology as a consequence of EHEC infection includes cortical necrosis, glomerular thromboses and congestion with widened subendothelial space, endothelial cell swelling, neutrophilia, and occasional mesangiolysis [46,47]. In the baboon model of Stx challenge which presents with all manifestations of HUS [48], glomerular injury after Stx1 and Stx2 challenge featured prominent HUS-like capillary wall changes, including thickening of the glomerular basement membrane and double contouring with prominent thrombus formation, and severe endothelial injury with diffuse cell swelling and focal endothelial denudation (Figure 3). Because this is an animal model allowing comparisons between toxins, some differences emerged with glomerular endothelial injury predominating after Stx1, whereas mesangiolysis and eosinophilic infiltration accompanied the glomerular injury in the Stx2-challenged animals [49]. 

Endothelial activation by the Shiga-like toxins induces an inflammatory milieu that is a strong contributor to the thrombi formation in the microvasculature during development of HUS. Stx2 upregulates chemokines monocyte chemotactic protein-1 (MCP-1, CCL2) and IL-8 (CCL8) [27,42] and Stx1 increases expression of cellular adhesion molecules ICAM-1, VCAM-1, and E-selectin on endothelial cells [50]. In human monocytes, EHEC infection and Shiga toxins induces inflammatory cytokines and chemokines including IL-1β, IL-6, IL-8, and TNFα [51]. In the GI of patients, the colon is severely affected, showing edema and hemorrhage, consistent with the hemorrhagic colitis that is often seen preceding HUS [52]. Intestinal leukocytosis is more prominent with D + HUS compared to non-EHEC HUS [53], and with Stx in the intestinal epithelial cells [9], there is likely production of pro-inflammatory and chemotactic mediators (IL-1, IL-6, IL-8, TNFα) from epithelial cells and resident macrophages [54,55,56]. Together, this group of chemokines and adhesion molecules serves to recruit and activate neutrophils, contributing to increased tissue damage. 

Normally endothelial cells present non-thrombogenic surfaces, and both Shiga toxins and inflammatory mediators can shift the endothelium to a pro-thrombotic state, contributing to thrombus formation [57]. For example, Stx2 causes about a 15% decrease in thrombomodulin surface antigen expression of human glomerular endothelial cells *in vitro*, which is further potentiated by TNFα [58], although whether this amount of antigen loss significantly impacted function was not determined. Thrombomodulin is an essential cofactor of the protein C anticoagulant pathway, an assembly of molecules on the endothelium that maintains blood fluidity [59,60]. Loss of thrombomodulin expression, or other members of this pathway, correlates with a pro-coagulant environment [61] and circulating soluble thrombomodulin levels are accepted measures of endothelial injury [62]. 

However, endothelial and leukocyte activation are probably only part of what induces the formation of microthrombi during D + HUS. Studies have also looked at whether platelet activation might contribute to microthrombi genesis. Plasma from patients with D + HUS has shown evidence of platelet activation and degranulation [63]. Stx1 can activate endothelial cells, especially ones that express more Gb_3_ receptor due to TNFα or LPS priming, to cause increased adhesion of platelets [64] and inhibition of platelet cluster degradation by the inhibition of ADAMTS13 [65]. Collectively, endothelial, platelet and neutrophil activation all contribute to the formation of microthrombi that then becomes a major driving force of D + HUS pathophysiology.

Cytokines can disrupt the anti-thrombotic properties of the microvascular endothelial cells and increase cell-surface Gb_3_ receptor expression [53]. Obrig *et al.* hypothesized previously that the sensitivity of endothelial cells to Shiga toxins depended greatly on their source and levels of Gb_3_ expression, and found that while TNFα and lipopolysaccharide (LPS) could increase sensitivity of human umbilical vein endothelial cells to Shiga toxins, they had no effect on renal microvascular endothelial cell Gb_3_ expression or Stx sensitivity [26]. It has been shown that the numbers of polymorphonuclear leukocytes present at the beginning of D + HUS is predictive of outcome, but not in cases of atypical HUS [66], a related but distinct syndrome (see below). Stx induces an inflammatory and chemotactic environment, and recruited neutrophils may transport the toxins in the blood [67], which could explain how the toxin passes from the gut to the kidney without bacteremia or invasion by the EHEC. Whether this occurs in patients is debated [68] because Stx antigen is sometimes [67,69,70], but not always [71] detected on the surface of circulating neutrophils. Close proximity of neutrophils and their enzymes to renal endothelial cells can also alter the surface availability of some proteins, changing the functionality of the endothelium. It is well known that neutrophil elastase cleaves endothelial-expressed thrombomodulin [72], a necessary cofactor of the protein C anticoagulant pathway [59]. If this proteolysis is incomplete, and removes only the amino-terminal lectin domain of thrombomodulin, this may contribute to organ injury and thrombotic microangiopathy, as suggested in murine models of Stx2 challenge with endotoxin priming [73]. 

It is interesting to note that while this information helps to explain the forces driving disease in the kidney and other organs, they do not go as far in explaining the neurological signs that are often seen accompanying HUS. In the recent 2011 German outbreak of EHEC infection, the rate of HUS was unusually high and the neurologic complications were severe, from aphasia to epileptic seizures. Notably, there were no histologically observed brain microthrombi, vessel occlusions or evidence of ischemia [74]. How then did the patients develop neurological symptoms, and was Stx to blame? One study in rats has shown that Stx1 effect on astrocytes could indirectly affect the blood-brain barrier [75]. During the German outbreak, immunoadsorption of IgG helped attenuate the neurological complications of Stx2-producing *E. coli* O104:H4 [76]. They did not find evidence that the antibodies were directed against ADAMTS13, loss of which contributes to a related thrombotic microangiopathy (see below), and instead hypothesized that the antibodies were against Stx itself and were crosslinking receptors, causing the release of larger von Willebrand Factor multimers [77] to indirectly inhibit ADAMTS13 [65] and increasing risk of thrombus formation. Further investigation will show whether this interesting hypothesis is true.

A potential role for the complement pathway during development of Stx-induced HUS has recently gained renewed interest. Complement is an ancient non-specific opsonic defense mechanism dating to pre-Cambrian ancestors [78], and is now a highly regulated mammalian innate immune strategy contributing to host defense or tissue injury depending on the context [79]. There is some evidence that complement is activated during EHEC infection. During acute phase EHEC-HUS, complement factor C3 or C9 is detected on platelet-leukocyte complexes from patients, and Stx can induce *ex vivo* release of complement-bearing microparticles from platelets or monocytes [80]. Using purified components, Stx2 was shown to activate complement via the alternative pathway and slow complement factor H (CFH) regulatory activity [81]. A recent study showed Stx-induced C3b interactions with P-selectin to promote a pro-thrombotic surface on dermal microvascular endothelial cells activated with human serum and/or blood under shear stress [82]. These observations, along with C3 deposition on glomeruli from mice challenged with Stx2 + LPS [82], suggest a role for complement in the pathophysiology of endothelial dysfunction during HUS. However, patient data has less apparent clarity. In a small patient study, increased levels of alternative pathway members Bb and SC5b-9 were observed early in D + HUS patients, but there was no correlation with clinical parameters or outcome [83]. There also was no comparison with non-EHEC renal patients so it is difficult to evaluate a specific relationship to Stx-HUS as opposed to a generalized host defense response. In addition, despite apparent success in three severely ill infants [84], treatment of 67 patients with Eculizumab (Soliris^®^, Alexion Pharmaceuticals), the anti-C5 inhibitor antibody treatment for atypical HUS [85], during the 2011 outbreak of Stx2-producing EHEC 0104:H4 in Germany was not effective and likely worsened patient outcome [21]. This was a severely ill cohort with other co-treatments so efficacy interpretation is debated [86,87], but this is likely the patient population that such a treatment would target. Given the fundamental contribution of the complement pathway to pathogenesis of many diseases, it could potentially contribute to disease severity, but whether it actually initiates HUS during this bacterial infection remains to be determined. 

## 4. Atypical Hemolytic Uremic Syndrome (Non-EHEC)

HUS not due to enteropathogenic bacterial infection is known as atypical HUS (aHUS) and there are many triggers besides infection, with the underlying cause being genetic deficiencies in regulators of the alternative pathway of complement [88]. In this case, the alternative pathway of complement is a fundamental initiator of disease. aHUS leads to recurring bouts of HUS, and can lead to stroke, heart complications and end-stage renal failure, although how quickly this progresses depends on the nature of mutation in the complement pathway members [89]. Histologically, aHUS is indistinguishable from D + HUS in the kidney. A majority (60%–70%) of patients with cofactor H (CFH) mutations progressed to end-stage renal failure within a year of aHUS symptoms [90]. Many children experience their first bout of aHUS before age 6 months, which can help lead a differential diagnosis towards aHUS instead of D + HUS [91]. Treatment with Eculizumab (Soliris^®^), a humanized monoclonal antibody against complement C5, has had success in aHUS patients because it blocks the rampant, dysregulated activation of C5 thereby preventing formation of inflammatory C5a and the cytotoxic terminal attack complex C5b-9 [85,92]. 

Atypical HUS can be sporadic or familial, and is associated with dysregulation of complement typically characterized either by over-activation or compromised regulators of the alternative pathway of complement. Ordinarily, complement factor H (CFH), along with complement factor I (CFI) and membrane cofactor protein (MCP) accelerates the decay of C3 convertase, inhibiting complement activation. Thrombomodulin, an endothelial cell surface membrane protein usually associated with anti-coagulant function [62], aids this enzymatic complex in its activity. Mutations in thrombomodulin [93] as well as CFH [94], CFI [90], and MCP [95] are all implicated as risk factors for development of aHUS, along with mutations in complement factor B [96] and C3 [97]. In addition, autoantibodies against CFH have also been demonstrated [89]. While infection can be a possible trigger for the manifestation of aHUS, in general there is much less evidence of leukocytosis and inflammation in patients with aHUS than in those with D + HUS [53]. Yet, there may be overlap and at least one patient with EHEC-HUS has been shown to also have a mutation in MCP that would confer risk of developing aHUS [98], emphasizing how the lines between these syndromes can be blurred. 

## 5. Thrombotic Thrombocytopenic Purpura

A third syndrome closely related to aHUS is thrombotic thrombocytopenic purpura (TTP), a thrombotic microangiopathy defined by a “pentad” consisting of the triad of HUS with accompanying fever and neurological manifestations [99]. Many HUS patients develop neurological symptoms and TTP patients can have kidney failure, so in many cases the diagnosis is given as HUS/TTP. A major risk factor for the development of TTP is a deficiency in the plasma von Willebrand factor-cleaving protease ADAMTS13, due to either a genetic etiology or autoimmune antibodies against the protease [99]. The resultant high levels of ultra-large vWF multimers in the circulation lead to increased platelet-containing thrombi. Although HUS, aHUS, and TTP are closely related diseases and often difficult to distinguish in the clinic, they do have separate causes as well as distinct outcomes. Because aHUS and TTP are due to autoantibodies, deficiencies or mutations of plasma factors, plasma exchange can be used to treat both. In addition, for aHUS, kidney or combined kidney-liver transplants have also shown to be beneficial when supplemented with plasma exchanges [100]. 

Recent studies have shown that the distinctions between TTP and D + HUS with respect to clinical signs and pathophysiology may be less defined based on the report by Motto *et al.* that Stx can trigger TTP in ADAMTS13-deficient mice although absence of ADAMTX13 alone is insufficient [77]. In addition, Stx1 or Stx2 causes the release of large vWF multimers from human endothelial cells, while also impairing ADAMTS13 activity, and LPS was shown to have minimal contribution [65]. Huang *et al.* later showed that the Stx B subunit was sufficient to induce vWF secretion and thrombotic microangiopathy in ADAMTS13^−/−^ mice back-bred for toxin susceptibility [77], something which had been shown for Shiga toxins earlier [101]. 

## 6. Animal Models

Though the first recorded US outbreak of enterohemorrhagic *Escherichia coli *occurred in 1982, a complete picture of how EHEC elicits disease in humans remains to be elucidated. EHEC infections are typically unpredictable common source outbreaks, so there are no endemic patient populations for systemic study of disease pathogenesis. The development of animal models has thus been necessary in order to study toxin-specific mechanisms that govern the observed disease process in humans. Animals may receive purified toxin(s) or bacteria, but despite the numerous animal models tried, none of them completely replicate the EHEC infection and HUS observed in patients. However, they do fulfill an important role in that they allow for the investigation of one or more aspects of pathogenesis of EHEC disease and HUS [102]. A brief summary of animal model features and limitations are shown in Table 1 with representative references. 

**Table 1:** Animal models.

Model	Pre-treatment	Animal	Features	Limitations	Reference
EHEC	none	Gnotobiotic piglet	Focal renal lesions, renal thrombotic microangiopathy	Normal serum creatinine; no thrombocytopenia	[103]
EHEC	streptomycin	CD-1 mice	Bacterial colonization; loose stools, anorexia, lethargy	No disease unless host-adapted strain used; no glomerular damage, coagulopathy or thrombocytopenia	[104,105]
EHEC	streptomycin and mitomycin C	ICR mice	Weakness, weight loss, microhemorrhages in brain and spinal cord, high BUN	Serum creatinine normal, unremarkable kidney pathology	[106]
EHEC	None or TNFα	Germ free IQI mice	Anorexia, renal tubular necrosis, thrombocytopenia, leukocytosis	No glomerular histopathology, inflammation only with TNFα pre-treatment	[107]
EHEC	24 hour fast	C3H/HeJ mice	Gastrointestinal, neurologic and systemic symptoms, renal inflammation and necrosis, fragmented red blood cells	Kidney function and platelets not measure	[108]
EHEC	none	Newly weaned BALB/c mice	Renal damage, high urea concentrations, colon pathology	Thrombotic microangiopathy not evaluated	[109]
EHEC	Protein calorie malnutrition	C57Bl/6J mice	Systemic and neurologic symptoms, increased BUN, mild renal tubular degeneration	Normal serum creatinine, normal glomeruli, no significant platelet changes	[110]
EHEC	Host-adapted bacteria	Dutch Belted rabbits	Diarrhea, lethargy, anorexia, dehydration glomerular thrombi and renal congestion	No consistent thrombocytopenia	[111]
Stx1	none	baboon	Thrombocytopenia, schistocytosis and hemolytic anemia, renal failure, GI injury, lesions in CNS, systemic inflammation	Non-bacterial	[42,48]
Stx2	none	baboon	Thrombocytopenia, leukocytosis, acute renal failure, schistocytosis, hemolytic uremia, glomerular thrombotic microangiopathy, systemic inflammation	Non-bacterial	[42,112]
Stx2	none	C57Bl/6J mice	Increased plasma BUN and creatinine, hemolysis, neutrophilia	Non-bacterial, no thrombocytopenia	[113]
Stx2+LPS	none	C57Bl/6J mice	Neutrophilia, thrombocytopenia, hemolysis, increased BUN and creatinine, renal histopathology	Non-bacterial, LPS effect depends on timing of administration, consumptive coagulopathy	[29]
EHEC culture supernatant	none	Sprague-Dawley rats	Increased BUN and creatinine, thrombocytopenia, hemolytic anemia and leukocytosis, renal histologic changes, watery diarrhea	Non-bacterial, crude bacterial supernatants rather than purified toxin(s).	[114]

## 7. Gnotobiotic Piglet Models

One of the earliest animal models of EHEC-mediated HUS was the gnotobiotic piglet. This animal had previously been used to study other human enteric infections [115]. Piglets were infected with ~1 × 10^10^ CFU *E. coli *O157:H7 twenty-four hours after delivery and were euthanized and necropsied on either day three or day five. Ulcers and mononuclear cell infiltration were observed in the cecums of the animals euthanized on day five. Only minor focal lesions associated with bacterial antigen were observed in the kidney, and no hematological symptoms of hemolytic uremic syndrome were described [116]. 

Gnotobiotic piglets were again used as an animal model for EHEC infection by Gunzer *et al.* in 2002, and in these experiments the course of disease resembled that of human EHEC infection in the GI tract as well as other organs. Renal histopathology changes included surface petechiae and diffuse glomerular endothelial swelling with mild to moderate tubular damage. Notably, renal thrombotic microangiopathy was observed in most animals. Limitations to the model can be found in the clinical chemistry and hematologic findings. Though individual animals had elevated serum creatinine levels, the averages were not statistically significant between the experimental groups and the control animals. Furthermore, thrombocytopenia was not observed, nor were schistocytes in peripheral blood smears [103]. Use of gnotobiotic piglets as a model for EHEC is thus useful in studies that focus on neurological or renal changes, but does not accurately mimic the full spectrum of HUS symptoms observed in humans. 

## 8. Murine EHEC Models

Relative to humans, mice are fairly resistant to EHEC infection, so murine models include stressors intended to increase susceptibility to intestinal bacterial colonization and infection. These approaches include pre-treatment with antibiotics, germ-free environments, and protein calorie malnutrition diets [117]. Antibiotics added to the animals’ drinking water reduces or alters the normal intestinal facultative bacteria, increasing the likelihood of EHEC intestinal colonization by decreasing bacterial competition. An early study used streptomycin to study the colonization abilities of several *E. coli *isolates [104] and Wadolkowski *et al.* adopted streptomycin pre-treatment to develop a murine model of EHEC infection [105]. Streptomycin-treated male CD-1 mice were infected with either wild-type *E. coli *O157:H7, a mutant strain that did not contain the plasmid encoding fimbriae, or both. Animals demonstrated colonization of the bacteria in all three conditions, though disease symptoms were not observed. However, mice inoculated with the mutant strain that had been previously recovered from the feces of one of the co-infection animals demonstrated symptoms of infection including loose stools, anorexia and lethargy between days 4–10 post-inoculation, with lethality following within a few days. Colonization without infection was the outcome unless the bacteria strain was host-adapted. The colons of these mice did not show gross or histological lesions, indicating that the animals did not die of hemorrhagic colitis. Rather, death appeared to be due to acute necrosis of proximal convoluted tubules, a lesion which is characteristic of toxin exposure in mice. However, the mice did not show evidence of glomerular damage, thrombotic microangiopathy, or thrombocytopenia [105]. 

It had been demonstrated previously that addition of mitomycin C to *in vitro *EHEC cultures resulted in induction of phage expression and increased toxin production [118,119]. Young male ICR mice were pre-treated with streptomycin and mitomycin, then infected with EHEC strain E32511/HSC (Stx2c variant) pre-selected to be resistant to both antibiotics. The animals developed weakness and weight loss, but not diarrhea [106]. Microhemorrhages were observed in the brain and spinal cord, as were slight degeneration of tubules in the kidney. Blood urea nitrogen was significantly higher in experimental mice than in controls, suggesting renal injury, but serum creatinine levels did not change and light microscopy kidney pathology examinations were unremarkable. Acute encephalopathy with loss of blood brain barrier function, brain endothelial cell edema, and demyelination were predominant histologic features. Toxin antigen was detected in affected neurons, but it was not clear whether the neurologic symptoms were from primary neuronal lesions or secondary to hypoxia from thrombotic microangiopathy. 

The rationale behind antibiotic pretreatment of mice can be extended to include germ-free mouse models of EHEC infection. Isogai *et al.* showed that infection with a high dose (2 × 10^9^ CFU) of *E. coli *O157:H7 was lethal within 7 days of inoculation for germ-free IQI mice. The mice became anorexic and urine retention was associated with renal tubular necrosis [107]. Interestingly, thrombocytopenia and leukocytosis were observed in infected animals. In order to elucidate a role for pro-inflammatory TNFα in EHEC pathogenesis, the authors included a group of mice pretreated with the cytokine. The addition of TNFα nearly doubled the lethality from 60% to 100% with the addition of neurologic symptoms. Furthermore, cytokine-treated mice demonstrated histopathologic changes in the kidney including proliferation of glomerular mesangial cells and microthrombosis, and increased cytokine responses in serum, kidney and brains. Based on these experiments, the authors concluded that the toxins produced by EHEC and/or bacterial endotoxin, induce production of TNFα to have an additive effect EHEC pathogenesis [107]. Priming of cells with pro-inflammatory mediators, especially TNFα, is well known to enhance subsequent cellular responses to pathogenic challenge, and this occurs both *in vitro* and *in vivo* [120]. Similarly, TNFα induced by bacterial endotoxin enhances subsequent Stx cytotoxicity in rat astrocytes and releases chemokines that may attract PMNs to contribute to neurological injury and loss of endothelial barrier function [121,122]. 

Though pre-treatment with antibiotics or the use of germ-free animals increases the likelihood of gut EHEC colonization and infection, it does not accurately depict the circumstance when normal gut flora is present as is the case with patients. To address this, alternative models were developed. C3H/HeJ mice have a TLR4 mutation, rendering them moderately immunocompromised and thus more susceptible to infection. These mice were fasted for 24 h prior to infection, and challenged with 1 × 10^9^ CFU/mL EHEC expressing Stx2 by oral gavage [108]. They developed gastrointestinal symptoms including loose, watery stools, neurological symptoms including ataxia and convulsions, and systemic symptoms including lethargy, anorexia and shivering with ~40% mortality. Histologic examination of colon tissue demonstrated inflammatory infiltrates and necrotic foci. In the kidneys, they observed proliferation of glomerular mesangial cells, diffuse interstitial inflammation and necrosis of tubular cells. In addition, the authors noted fragmented red blood cells in the sick mice. Blood samples were not analyzed for markers of kidney function or for platelet numbers, so a full description of HUS pathology was not available [108].

Newly weaned BALB/c mice showed increased susceptibility to EHEC infection and Stx-mediated renal damage without pretreatment to alter commensal flora [109]. Between 22% and 27% of infected young animals developed high urea concentrations in the serum and all of these mice died. Mortality was associated with increased circulating neutrophils, followed by a significant decrease in all circulating leukocytes shortly before death. Colon pathology included leukocyte infiltration, and the kidney showed focal cortical necrosis with tubular epithelial swelling, although thrombotic microangiopathy was not evaluated. 

To initiate EHEC colonization and infection, murine models require much higher bacteria doses compared to humans (murine ~10^9^ CFU *versus* 10–100 organisms for humans). The observation that a subset of children infected with EHEC had an unbalanced diet prior to infection inspired Kurioka *et al.* to hypothesize that protein calorie malnourished (PCM) mice would contract disease from lower doses of EHEC than conventional mice. Indeed, the minimal infectious dose of *E. coli* in the PCM mice was lower at 2 × 10^6^ CFU. [110]. The infected PCM mice developed systemic and neurologic symptoms, no gastrointestinal symptoms were observed. BUN levels, but not creatinine, increased and acute kidney injury was minimal with mild renal tubular degeneration and normal glomeruli. Significant platelet changes were not observed [110]. The advantage of the PCM murine model is the lower inoculum dose with neurologic complications, but the animals do not appear to develop HUS. 

## 9. Rabbit EHEC Model

Dutch Belted rabbits orally infected with 5–9 × 10^8^ CFU of host-adapted EHEC O103 (Stx1+ Stx2-) or patient-derived O157:H7 (Stx1+ Stx2+) developed renal vascular lesions and glomerular lesions containing erythrocyte fragments [111]. These animals also demonstrated diarrhea, lethargy, anorexia, dehydration and weight loss with a low hematocrit observed in five of six infected animals. Similar to that seen in humans, glomerular, perivascular and tubular injury was observed in all animals after challenge with either strain, as was glomerular endothelial edema, mesangial deposits and fibrin thrombi occluding capillary lumens. Lack of changes in creatinine suggests renal injury rather than renal insufficiency, and although mild to moderate thrombocytopenia was observed in only 3 of 30 experimental animals, glomerular thrombi and renal congestion suggest the presence of coagulopathy if not a full thrombotic microangiopathy. Intestinal edema and vascular lesions with colonic inflammation with leukocyte infiltration was observed, as was surface-adherent *E. coli* in the cecal mucosa and crypt lumens. This animal model is a reasonable mimic of the human renal and intestinal lesions induced by EHEC and its toxins, although lack of consistent thrombocytopenia limits this model with respect to recapitulating human HUS. 

## 10. Nonhuman Primate Toxin Models of HUS

Several animal models examine the effects of purified Shiga toxin, in the absence of bacteria, with variable success in mimicking human HUS symptoms. A descriptive study of a baboon model of Shiga toxin 1 responses observed renal failure with death in 57 h or less, and the animals presented, for the first time, with all the classic clinical symptoms of HUS [48]. Animals in the low-dose Shiga toxin 1 group (50–200 ng/kg i.v.; *n* = 8) demonstrated thrombocytopenia, schistocytosis and hemolytic anemia, renal injury with glomerular endothelial injury and an inflammatory profile, renal failure, and injury to the GI tract. Lesions in the central nervous system were observed by electron microscopy. Stearns-Kurosawa *et al.* expanded the baboon model to include dose-response studies of both toxins for comparative purposes [42]. Stx2-induced renal injury and mortality was delayed 2–3 days compared to Stx1 challenge, and animals were ultimately more sensitive to Stx2, succumbing at lower doses (50 ng/kg for Stx2 *versus* 100 ng/kg Stx1). Both toxins elicited a systemic inflammatory response, but levels of cytokines and chemokines were elevated earlier and to higher levels after Stx1 challenge. Care was taken to ensure low endotoxin contamination of the toxins, and TNFα was not detectable in either plasma or urine from any of the animals. If pro-inflammatory priming is a necessary event for Stx toxicity, then it may be more complicated than production of TNFα. Unlike other animal models, the development of thrombocytopenia was consistently observed and was toxin dose-dependent. Loss of platelets was more gradual in Stx2-challenged animals as compared to Stx1, however anemia development was comparable and long-lasting [112]. Challenge with either toxin resulted in renal failure, increased plasma blood urea nitrogen (BUN) and creatinine, and deteriorating urinalysis profiles. The baboons could be rescued from lethal Stx2 challenge with a custom synthetic peptide designed to counter Stx2 activity within cells [112]. Renal function and urine output was preserved even when the peptide was administered 24 h after the otherwise lethal Stx2 challenge. Thus, both Stx1 and Stx2 are capable of eliciting HUS in baboons, but the timing and magnitude of the responses differ.

## 11. Rodent Models of Stx Challenge

Although the pathology of human HUS can be mimicked in nonhuman primates through the administration of Stx alone, a small animal model of HUS would provide many benefits and be accessible to more investigators. 

C67BL/6J mice, when given only multiple sub-lethal doses of Stx2 and no pre-treatments, develop some but not all aspects of human HUS [113]. Significantly increased plasma BUN and creatinine levels and proteinuria reflect glomerular renal injury. Hemolysis, neutrophilia and lymphocytopenia were observed. Histologically, the kidneys showed fibrin (ogen) deposition in the glomerular capillary loops and swollen subendothelial zones containing flocculent material [113]. Thrombocytopenia, however, was not observed and lack of this HUS symptom appears to be common to all Stx murine models unless co-treated with an accessory pro-inflammatory mediator. 

Modeling HUS using mice challenged with EHEC toxins has been described using co-administration of Gram negative endotoxin (a.k.a., lipopolysaccharide, LPS) and Stx2 [123], a model that has been used recently by multiple investigators. This approach was based on the hypothesis that LPS from the commensal flora of the gut could potentially contribute to the pathogenesis of HUS. However, LPS had either a synergistic or protective effect, depending on the timing of administration. Keepers *et al.* found that when C57BL/6 mice were given low sublethal doses of both LPS and Stx2 concurrently, they exhibited all signs of clinical HUS [29]. Neutrophilia, thrombocytopenia, red cell hemolysis and increases in serum creatinine and BUN were all observed. In the kidney, glomerular fibrin deposition, microthrombi formation and glomerular ultrastructure changes were demonstrated by histology and electron microscopy [29]. 

Priming of the Stx system in this way with LPS, TNFα, ADP or similar potentiates toxin chemotactic and inflammatory responses *in vitro* and *in vivo* [29,117], but the relevance to disease pathogenesis in patients is not clear. In experimental settings, addition of LPS priming to Stx2 challenge in mice will induce thrombocytopenia, but this etiology is a consumptive disseminated intravascular coagulation [124,125] rather than HUS and they have different coagulation profiles. This differentiation was discussed by Karmali *et al.* decades ago [17]. Additionally, the notion that compromised intestinal barrier function allows bacterial translocation or LPS leakage from commensal or pathogenic bacteria is far from established. This theory has been discussed with variable levels of support since the 1940s with respect to bacterial sepsis mechanisms and is proven to be poorly related to the patient condition, particularly for endotoxemia [126]. Some patients with EHEC O157:H7 infection have antibodies (usually IgM or IgA) against the bacterial LPS serotype [127,128] but detection in healthy control sera as well as cross-reactivity with other LPS serotypes adds complexity [129], and there is no prospective evidence showing these antibodies are protective or prognostic. In non-EHEC bacterial sepsis, circulating *E. coli* LPS is detectable in patients, but targeted neutralization of Gram negative LPS has consistently failed to improve 28 day all-cause mortality [130,131]. The current thinking is that the primary contribution of the intestine during these infections comes from host cellular injury with resultant host-derived mediators to propagate local and systemic inflammation effects [132,133]. 

Rats were studied as a Shiga toxemia model by Zotta *et al.* in 2008 to evaluate their ability to mimic development of HUS. Adult male Sprague-Dawley rats were challenged intraperitoneally with increasing volumes of filtered EHEC culture supernatant containing Stx2, but low endotoxin, and disease progression was monitored for 48 h. At an estimated toxin dose of 20 μg Stx2/kg body weight, all animals developed increased BUN and creatinine, thrombocytopenia (decreased from 84 × 10^6^/μL to 33 × 10^6^/μL), hemolytic anemia and leukocytosis [114], clinical markers that correlate with disease progression in patients [2,18]. Histological observations included necrotic glomerular areas, tubular injury, and thrombotic microangiopathy. Watery diarrhea due to colonic mucosa damage was also observed, but hemorrhagic colitis was not a feature. This methodology provides a small animal model of HUS that effectively reproduces the pathology of human HUS. However, this model has not been used frequently, likely because it used crude bacterial supernatants rather than purified toxin. Bacterial virulence factors other than Stx may contribute to disease pathophysiology [134] and the observation that crude bacterial supernatants, but not purified Stx, can induce HUS in rodents is consistent with this. These presumed non-Stx virulence factors bear further investigation. 

## 12. Conclusions

In the pursuit of effective translational medicine for EHEC infection, animal models that accurately mimic the pathogenesis of EHEC-induced human HUS are crucial if we are to fully elucidate the mechanisms involved. A plethora of such models have been spurned by this need, however none encapsulate the pathogenesis of EHEC infection and Shiga toxins-induced human HUS in its entirety. Nonetheless, each model provides certain insights, and may ultimately help the scientific community progress in its understanding of this disease. From these models and patient studies, identification of biomarkers that discriminate related clinical syndromes, quantify the early onset or risk of HUS, and report patient physiological responses to clinical intervention will ultimately prove to be of greatest value. 

## Appendix

Expanded *post-hoc* analysis data presented at the November 2012 meeting of the American Society of Nephrology by Dr. Rolf Stahl (University Hospital Hamburg-Eppendorf, Hamburg, Germany) provided very promising and strong evidence for efficacy of Eculizumab (anti-complement C5; Alexion) in their severely ill patient cohort during the recent EHEC O104:H4 outbreak. Data showing more rapid recovery of platelet counts with resolution of acute kidney injury and neurological symptoms compared with a clinically comparable cohort not treated with antibody was presented and is under peer review for publication. This gives renewed optimism that targeting complement pathways may be beneficial for patients with EHEC infection.

## References

[1] Riley L.W., Remis R.S., Helgerson S.D., McGee H.B., Wells J.G., Davis B.R., Hebert R.J., Olcott E.S., Johnson L.M., Hargrett N.T. (1983). Hemorrhagic colitis associated with a rare *Escherichia coli* serotype. N. Engl. J. Med..

[2] Bell B.P., Goldoft M., Griffin P.M., Davis M.A., Gordon D.C., Tarr P.I., Bartleson C.A., Lewis J.H., Barrett T.J., Wells J.G. (1994). A multistate outbreak of *Escherichia coli* O157: H7-associated bloody diarrhea and hemolytic uremic syndrome from hamburgers. The Washington experience. J. Am. Med. Assoc..

[3] Rangel J.M., Sparling P.H., Crowe C., Griffin P.M., Swerdlow D.L. (2005). Epidemiology of *Escherichia coli* O157:H7 outbreaks, United States, 1982-2002. Emerg. Infect. Dis..

[4] Schmidt H., Geitz C., Tarr P.I., Frosch M., Karch H. (1999). Non-O157:H7 pathogenic Shiga toxin-producing *Escherichia coli*: Phenotypic and genetic profiling of virulence traits and evidence for clonality. J. Infect. Dis..

[5] Beutin L., Zimmermann S., Gleier K. (1998). Human infections with Shiga toxin-producing *Escherichia coli* other than serogroup O157 in germany. Emerg. Infect. Dis..

[6] Savage P.J., Campellone K.G., Leong J.M. (2007). Interaction of enterohemorrhagic *Escherichia coli* (EHEC) with mammalian cells: Cell adhesion, type iii secretion, and actin pedestal formation. Curr. Protoc. Microbiol..

[7] Farfan M.J., Torres A.G. (2012). Molecular mechanisms that mediate colonization of Shiga toxin-producing *Escherichia coli* strains. Infect. Immun..

[8] Melton-Celsa A., Mohawk K., Teel L., O’Brien A. (2012). Pathogenesis of Shiga-toxin producing *Escherichia coli*. Curr. Top Microbiol. Immunol..

[9] Malyukova I., Murray K.F., Zhu C., Boedeker E., Kane A., Patterson K., Peterson J.R., Donowitz M., Kovbasnjuk O. (2009). Macropinocytosis in Shiga toxin 1 uptake by human intestinal epithelial cells and transcellular transcytosis. Am. J. Physiol. Gastrointest. Liver Physiol..

[10] Gould L.H., Demma L., Jones T.F., Hurd S., Vugia D.J., Smith K., Shiferaw B., Segler S., Palmer A., Zansky S. (2009). Hemolytic uremic syndrome and death in persons with *Escherichia coli* O157:H7 infection, foodborne diseases active surveillance network sites, 2000-2006. Clin. Infect. Dis..

[11] Fukushima H., Hashizume T., Morita Y., Tanaka J., Azuma K., Mizumoto Y., Kaneno M., Matsuura M., Konma K., Kitani T. (1999). Clinical experiences in Sakai City hospital during the massive outbreak of enterohemorrhagic *Escherichia coli* O157 infections in Sakai City, 1996. Pediatr. Int..

[12] Frank C., Faber M., Askar M., Bernard H., Fruth A., Gilsdorf A., Hohle M., Karch H., Krause G., Prager R. (2011). Large and ongoing outbreak of haemolytic uraemic syndrome, Germany, May 2011. Euro. Surveill..

[13] Pan D., Das A., Liu D., Veazey R.S., Pahar B. (2012). Isolation and characterization of intestinal epithelial cells from normal and SIV-infected rhesus macaques. PLoS One.

[14] Bielaszewska M., Mellmann A., Zhang W., Köck R., Fruth A., Bauwens A., Peters G., Karch H. (2011). Characterisation of the *Escherichia coli* strain associated with an outbreak of haemolytic uraemic syndrome in Germany, 2011: A microbiological study. Lancet Infect. Dis..

[15] Frank C., Werber D., Cramer J.P., Askar M., Faber M., an der Heiden M., Bernard H., Fruth A., Prager R., Spode A. (2011). Epidemic profile of Shiga-toxin-producing *Wscherichia coli* O104:H4 outbreak in Germany-Preliminary report. N. Engl. J. Med..

[16] Karmali M.A., Petric M., Lim C., Fleming P.C., Steele B.T. (1983). *Escherichia coli* cytotoxin, haemolytic-uraemic syndrome, and haemorrhagic colitis. Lancet.

[17] Karmali M.A., Petric M., Lim C., Fleming P.C., Arbus G.S., Lior H. (1985). The association between idiopathic hemolytic uremic syndrome and infection by verotoxin-producing *Escherichia coli*. J. Infect. Dis..

[18] Wong C.S., Mooney J.C., Brandt J.R., Staples A.O., Jelacic S., Boster D.R., Watkins S.L., Tarr P.I. (2012). Risk factors for the hemolytic uremic syndrome in children infected with *Escherichia coli* O157:H7: A multivariable analysis. Clin. Infect. Dis..

[19] Kimmitt P.T., Harwood C.R., Barer M.R. (2000). Toxin gene expression by Shiga toxin-producing *Escherichia coli*: The role of antibiotics and the bacterial SOS response. Emerg. Infect. Dis..

[20] Buchholz U., Bernard H., Werber D., Bohmer M.M., Remschmidt C., Wilking H., Deleré Y., an der Heiden M., Adlhoch C., Dreesman J. (2011). German outbreak of *Escherichia coli* O104:H4 associated with sprouts. N. Engl. J. Med..

[21] Menne J., Nitschke M., Stingele R., Abu-Tair M., Beneke J., Bramstedt J., Bremer J.P., Brunkhorst R., Busch V., Dengler R. (2012). Validation of treatment strategies for enterohaemorrhagic *Escherichia coli* O104:H4 induced haemolytic uraemic syndrome: Case-control study. Br. Med. J..

[22] Corogeanu D., Willmes R., Wolke M., Plum G., Utermohlen O., Kronke M. (2012). Therapeutic concentrations of antibiotics inhibit Shiga toxin release from enterohemorrhagic *E. coli* O104:H4 from the 2011 German outbreak. BMC Microbiol..

[23] Rivero M.A., Passucci J.A., Rodriguez E.M., Signorini M.L., Tarabla H.D., Parma A.E. (2011). Factors associated with sporadic verotoxigenic *Escherichia coli* infection in children with diarrhea from the central eastern area of Argentina. Foodborne Pathog. Dis..

[24] Garg A.X., Suri R.S., Barrowman N., Rehman F., Matsell D., Rosas-Arellano M.P., Salvadori M., Haynes R.B., Clark W.F. (2003). Long-term renal prognosis of diarrhea-associated hemolytic uremic syndrome: A systematic review, meta-analysis, and meta-regression. JAMA.

[25] Oakes R.S., Kirkham J.K., Nelson R.D., Siegler R.L. (2008). Duration of oliguria and anuria as predictors of chronic renal-related sequelae in post-diarrheal hemolytic uremic syndrome. Pediatr. Nephrol..

[26] Obrig T.G., Louise C.B., Lingwood C.A., Boyd B., Barley-Maloney L., Daniel T.O. (1993). Endothelial heterogeneity in Shiga toxin receptors and responses. J. Biol. Chem..

[27] Zoja C., Angioletti S., Donadelli R., Zanchi C., Tomasoni S., Binda E., Imberti B., te Loo M., Monnens L., Remuzzi G. (2002). Shiga toxin-2 triggers endothelial leukocyte adhesion and transmigration via NF-κb dependent up-regulation of IL-8 and MCP-1. Kidney Int..

[28] Reitsma P.H., Versteeg H.H., Middeldorp S. (2012). Mechanistic view of risk factors for venous thromboembolism. Arterioscler. Thromb. Vasc. Biol..

[29] Keepers T.R., Psotka M.A., Gross L.K., Obrig T.G. (2006). A murine model of HUS: Shiga toxin with lipopolysaccharide mimics the renal damage and physiologic response of human disease. J. Am. Soc. Nephrol..

[30] Camerer E., Cornelissen I., Kataoka H., Duong D.N., Zheng Y.-W., Coughlin S.R. (2006). Roles of protease-activated receptors in a mouse model of endotoxemia. Blood.

[31] Constantinescu A.R., Bitzan M., Weiss L.S., Christen E., Kaplan B.S., Cnaan A., Trachtman H. (2004). Non-enteropathic hemolytic uremic syndrome: Causes and short-term course. Am. J. Kidney Dis..

[32] Banerjee R., Hersh A.L., Newland J., Beekmann S.E., Polgreen P.M., Bender J., Shaw J., Copelovitch L., Kaplan B.S., Shah S.S. (2011). Streptococcus pneumoniae-associated hemolytic uremic syndrome among children in North America. Pediatr. Infect. Dis. J..

[33] Noris M., Remuzzi G. (2005). Hemolytic uremic syndrome. J. Am. Soc. Nephrol..

[34] Obrig T.G. (2010). *Escherichia coli* Shiga toxin mechanisms of action in renal disease. Toxins.

[35] Karch H., Tarr P.I., Bielaszewska M. (2005). Enterohaemorrhagic *Escherichia coli* in human medicine. Int. J. Med. Microbiol..

[36] Paton J.C., Paton A.W. (1998). Pathogenesis and diagnosis of Shiga toxin-producing *Escherichia coli* infections. Clin. Microbiol. Rev..

[37] O’Brien A.O., Lively T.A., Chen M.E., Rothman S.W., Formal S.B. (1983). *Escherichia coli* O157:H7 strains associated with haemorrhagic colitis in the United States produce a shigella dysenteriae 1 (Shiga) like cytotoxin. Lancet.

[38] Okuda T., Tokuda N., Numata S., Ito M., Ohta M., Kawamura K., Wiels J., Urano T., Tajima O., Furukawa K. (2006). Targeted disruption of Gb3/CD77 synthase gene resulted in the complete deletion of globo-series glycosphingolipids and loss of sensitivity to verotoxins. J. Biol. Chem..

[39] Saxena S., O’Brien A., Ackerman E. (1989). Shiga toxin, Shiga-like toxin II variant, and ricin are all single-site RNA *N*-glycosidases of 28S RNA when microinjected into Xenopus oocytes. J. Biol. Chem..

[40] Mukhopadhyay S., Linstedt A.D. (2012). Manganese blocks intracellular trafficking of Shiga toxin and protects against Shiga toxicosis. Science.

[41] Proulx F., Turgeon J.P., Litalien C., Mariscalco M.M., Robitaille P., Seidman E. (1998). Inflammatory mediators in *Escherichia coli* O157:H7 hemorrhagic colitis and hemolytic-uremic syndrome. Pediatr. Infect. Dis. J..

[42] Stearns-Kurosawa D.J., Collins V., Freeman S., Tesh V.L., Kurosawa S. (2010). Distinct physiologic and inflammatory responses elicited in baboons after challenge with Shiga toxin type 1 or 2 from enterohemorrhagic *Escherichia coli*. Infect. Immun..

[43] Smith W.E., Kane A.V., Campbell S.T., Acheson D.W.K., Cochran B.H., Thorpe C.M. (2003). Shiga toxin 1 triggers a ribotoxic stress response leading to p38 and JNK activation and induction of apoptosis in intestinal epithelial cells. Infect. Immun..

[44] Lee S.-Y., Lee M.-S., Cherla R.P., Tesh V.L. (2008). Shiga toxin 1 induces apoptosis through the endoplasmic reticulum stress response in human monocytic cells. Cell. Microbiol..

[45] Karpman D., Håkansson A., Perez M.T., Isaksson C., Carlemalm E., Caprioli A., Svanborg C. (1998). Apoptosis of renal cortical cells in the hemolytic-uremic syndrome: *In vivo* and *in vitro* studies. Infect. Immun..

[46] Inward C.D., Howie A.J., Fitzpatrick M.M., Rafaat F., Milford D.V., Taylor C.M. (1997). Renal histopathology in fatal cases of diarrhoea-associated haemolytic uraemic syndrome. Pediatr. Nephrol.

[47] Chaisri U., Nagata M., Kurazono H., Horie H., Tongtawe P., Hayashi H., Watanabe T., Tapchaisri P., Chongsa-nguan M., Chaicumpa W. (2001). Localization of Shiga toxins of enterohaemorrhagic *Escherichia coli* in kidneys of paediatric and geriatric patients with fatal haemolytic uraemic syndrome. Microb. Pathog..

[48] Taylor F.B., Tesh V.L., DeBault L., Li A., Chang A.C., Kosanke S.D., Pysher T.J., Siegler R.L. (1999). Characterization of the baboon responses to Shiga-like toxin: Descriptive study of a new primate model of toxic responses to Stx-1. Am. J. Pathol..

[49] Stearns-Kurosawa D.J., Oh S.-Y., Cherla R.P., Lee M.-S., Tesh V.L., Papin J., Henderson J., Kurosawa S. (2012). Distinct renal pathology and a chemotactic phenotype after enterohemorrhagic *E. coli* Shiga toxins.

[50] Morigi M., Micheletti G., Figliuzzi M., Imberti B., Karmali M.A., Remuzzi A., Remuzzi G., Zoja C. (1995). Verotoxin-1 promotes leukocyte adhesion to cultured endothelial cells under physiologic flow conditions. Blood.

[51] Van Setten P.A., Monnens L.A., Verstraten R.G., van den Heuvel L.P., van Hinsbergh V.W. (1996). Effects of verocytotoxin-1 on nonadherent human monocytes: Binding characteristics, protein synthesis, and induction of cytokine release. Blood.

[52] Richardson S.E., Karmali M.A., Becker L.E., Smith C.R. (1988). The histopathology of the hemolytic uremic syndrome associated with verocytotoxin-producing *Escherichia coli* infections. Hum. Pathol..

[53] King A.J. (2002). Acute inflammation in the pathogenesis of hemolytic-uremic syndrome. Kidney Int..

[54] Thorpe C.M., Hurley B.P., Lincicome L.L., Jacewicz M.S., Keusch G.T., Acheson D.W. (1999). Shiga toxins stimulate secretion of interleukin-8 from intestinal epithelial cells. Infect. Immun..

[55] Tesh V.L., Ramegowda B., Samuel J.E. (1994). Purified Shiga-like toxins induce expression of proinflammatory cytokines from murine peritoneal macrophages. Infect. Immun..

[56] Thorpe C.M., Smith W.E., Hurley B.P., Acheson D.W.K. (2001). Shiga toxins induce, superinduce, and stabilize a variety of C-X-C chemokine mRNAs in intestinal epithelial cells, resulting in increased chemokine expression. Infect. Immun..

[57] Zoja C., Buelli S., Morigi M. (2010). Shiga toxin-associated hemolytic uremic syndrome: Pathophysiology of endothelial dysfunction. Pediatr. Nephrol..

[58] Fernandez G.C., te Loo M.W., van der Velden T.J., van der Heuvel L.P., Palermo M.S., Monnens L.L. (2003). Decrease of thrombomodulin contributes to the procoagulant state of endothelium in hemolytic uremic syndrome. Pediatr. Nephrol..

[59] Weiler H. (2010). Regulation of inflammation by the protein c system. Crit. Care Med..

[60] Esmon C.T. (2005). The interactions between inflammation and coagulation. Br. J. Haematol..

[61] Lin S.M., Wang Y.M., Lin H.C., Lee K.Y., Huang C.D., Liu C.Y., Wang C.H., Kuo H.P. (2008). Serum thrombomodulin level relates to the clinical course of disseminated intravascular coagulation, multiorgan dysfunction syndrome, and mortality in patients with sepsis. Crit. Care Med..

[62] Kurosawa S., Stearns-Kurosawa D.J., Kinasewitz G.T. (2008). Soluble thrombomodulin: A sign of bad times. Crit. Care Med..

[63] Karpman D., Manea M., Vaziri-Sani F., Stahl A.L., Kristoffersson A.C. (2006). Platelet activation in hemolytic uremic syndrome. Semin. Thromb. Hemost..

[64] Morigi M., Galbusera M., Binda E., Imberti B., Gastoldi S., Remuzzi A., Zoja C., Remuzzi G. (2001). Verotoxin-1-induced up-regulation of adhesive molecules renders microvascular endothelial cells thrombogenic at high shear stress. Blood.

[65] Nolasco L.H., Turner N.A., Bernardo A., Tao Z., Cleary T.G., Dong J., Moake J.L. (2005). Hemolytic uremic syndrome-associated Shiga toxins promote endothelial-cell secretion and impair ADAMTS13 cleavage of unusually large von willebrand factor multimers. Blood.

[66] Walters M.D., Matthei I.U., Kay R., Dillon M.J., Barratt T.M. (1989). The polymorphonuclear leucocyte count in childhood haemolytic uraemic syndrome. Pediatr. Nephrol..

[67] Te Loo D.M., Monnens L.A., der Velden T.J., Vermeer M.A., Preyers F., Demacker P.N., van den Heuvel L.P., van Hinsbergh V.W. (2000). Binding and transfer of verocytotoxin by polymorphonuclear leukocytes in hemolytic uremic syndrome. Blood.

[68] Brigotti M. (2012). The interactions of human neutrophils with Shiga toxins and related plant toxins: Danger or safety?. Toxins.

[69] Tazzari P.L., Ricci F., Carnicelli D., Caprioli A., Tozzi A.E., Rizzoni G., Conte R., Brigotti M. (2004). Flow cytometry detection of Shiga toxins in the blood from children with hemolytic uremic syndrome. Cytometry B Clin. Cytom..

[70] Brigotti M., Tazzari P.L., Ravanelli E., Carnicelli D., Rocchi L., Arfilli V., Scavia G., Minelli F., Ricci F., Pagliaro P. (2011). Clinical relevance of Shiga toxin concentrations in the blood of patients with hemolytic uremic syndrome. Pediatr. Infect. Dis. J..

[71] Geelen J.M., van der Velden T.J.A.M., te Loo D.M.W.M., Boerman O.C., van den Heuvel L.P.W.J., Monnens L.A.H. (2007). Lack of specific binding of Shiga-like toxin (verocytotoxin) and non-specific interaction of Shiga-like toxin 2 antibody with human polymorphonuclear leucocytes. Nephrol. Dial. Transplant..

[72] Abe H., Okajima K., Okabe H., Takatsuki K., Binder B.R. (1994). Granulocyte proteases and hydrogen peroxide synergistically inactivate thrombomodulin of endothelial cells *in vitro*. J. Lab. Clin. Med..

[73] Zoja C., Locatelli M., Pagani C., Corna D., Zanchi C., Isermann B., Remuzzi G., Conway E.M., Noris M. Lack of the lectin-like domain of thrombomodulin worsens Shiga toxin-associated hemolytic uremic syndrome in mice. J. Immunol..

[74] Magnus T., Rother J., Simova O., Meier-Cillien M., Repenthin J., Moller F., Gbadamosi J., Panzer U., Wengenroth M., Hagel C. (2012). The neurological syndrome in adults during the 2011 northern German *E. coli* serotype O104:H4 outbreak. Brain.

[75] Landoni V.I., Schierloh P., de Campos Nebel M., Fernandez G.C., Calatayud C., Lapponi M.J., Isturiz M.A. (2012). Shiga toxin 1 induces on lipopolysaccharide-treated astrocytes the release of tumor necrosis factor-alpha that alter brain-like endothelium integrity. PLoS Pathog..

[76] Greinacher A., Friesecke S., Abel P., Dressel A., Stracke S., Fiene M., Ernst F., Selleng K., Weissenborn K., Schmidt B.M. (2011). Treatment of severe neurological deficits with igg depletion through immunoadsorption in patients with *Escherichia coli* O104:H4-associated haemolytic uraemic syndrome: A prospective trial. Lancet.

[77] Huang J., Motto D.G., Bundle D.R., Sadler J.E. (2010). Shiga toxin B subunits induce vWF secretion by human endothelial cells and thrombotic microangiopathy in ADAMTS13-deficient mice. Blood.

[78] Zhu Y., Thangamani S., Ho B., Ding J.L. (2005). The ancient origin of the complement system. EMBO J..

[79] Sarma J.V., Ward P.A. (2011). The complement system. Cell Tissue Res..

[80] Stahl A.L., Sartz L., Karpman D. (2011). Complement activation on platelet-leukocyte complexes and microparticles in enterohemorrhagic *Escherichia coli*-induced hemolytic uremic syndrome. Blood.

[81] Orth D., Khan A.B., Naim A., Grif K., Brockmeyer J., Karch H., Joannidis M., Clark S.J., Day A.J., Fidanzi S. (2009). Shiga toxin activates complement and binds factor H: Evidence for an active role of complement in hemolytic uremic syndrome. J. Immunol..

[82] Morigi M., Galbusera M., Gastoldi S., Locatelli M., Buelli S., Pezzotta A., Pagani C., Noris M., Gobbi M., Stravalaci M. (2011). Alternative pathway activation of complement by Shiga toxin promotes exuberant C3a formation that triggers microvascular thrombosis. J. Immunol..

[83] Thurman J.M., Marians R., Emlen W., Wood S., Smith C., Akana H., Holers V.M., Lesser M., Kline M., Hoffman C. (2009). Alternative pathway of complement in children with diarrhea-associated hemolytic uremic syndrome. Clin. J. Am. Soc. Nephrol..

[84] Lapeyraque A.L., Malina M., Fremeaux-Bacchi V., Boppel T., Kirschfink M., Oualha M., Proulx F., Clermont M.J., Le Deist F., Niaudet P. (2011). Eculizumab in severe Shiga-toxin-associated HUS. N. Engl. J. Med..

[85] Nurnberger J., Philipp T., Witzke O., Opazo Saez A., Vester U., Baba H.A., Kribben A., Zimmerhackl L.B., Janecke A.R., Nagel M. (2009). Eculizumab for atypical hemolytic-uremic syndrome. N. Engl. J. Med..

[86] Artunc F. (2012). Treating Shiga toxin induced haemolytic uraemic syndrome. Br. J. Haematol..

[87] Ruggenenti P., Remuzzi G. (2012). Thrombotic microangiopathy: *E. coli* O104:H4 German outbreak: A missed opportunity. Nat. Rev. Nephrol..

[88] Noris M., Caprioli J., Bresin E., Mossali C., Pianetti G., Gamba S., Daina E., Fenili C., Castelletti F., Sorosina A. (2010). Relative role of genetic complement abnormalities in sporadic and familial aHUS and their impact on clinical phenotype. Clin. J. Am. Soc. Nephrol..

[89] Kavanagh D., Goodship T. (2010). Genetics and complement in atypical HUS. Pediatr. Nephrol..

[90] Caprioli J., Noris M., Brioschi S., Pianetti G., Castelletti F., Bettinaglio P., Mele C., Bresin E., Cassis L., Gamba S. (2006). Genetics of HUS: The impact of MCP, CFH, and IF mutations on clinical presentation, response to treatment, and outcome. Blood.

[91] Loirat C., Fremeaux-Bacchi V. (2011). Atypical hemolytic uremic syndrome. Orphanet J. Rare Dis..

[92] Gruppo R.A., Rother R.P. (2009). Eculizumab for congenital atypical hemolytic-uremic syndrome. N. Engl. J. Med..

[93] Delvaeye M., Noris M., de Vriese A., Esmon C.T., Esmon N.L., Ferrell G., Del-Favero J., Plaisance S., Claes B., Lambrechts D. (2009). Thrombomodulin mutations in atypical hemolytic-uremic syndrome. N. Engl. J. Med..

[94] Warwicker P., Goodship T.H., Donne R.L., Pirson Y., Nicholls A., Ward R.M., Turnpenny P., Goodship J.A. (1998). Genetic studies into inherited and sporadic hemolytic uremic syndrome. Kidney Int..

[95] Richards A., Kathryn Liszewski M., Kavanagh D., Fang C.J., Moulton E., Fremeaux-Bacchi V., Remuzzi G., Noris M., Goodship T.H., Atkinson J.P. (2007). Implications of the initial mutations in membrane cofactor protein (MCP; CD46) leading to atypical hemolytic uremic syndrome. Mol. Immunol..

[96] Goicoechea de Jorge E., Harris C.L., Esparza-Gordillo J., Carreras L., Arranz E.A., Garrido C.A., Lopez-Trascasa M., Sanchez-Corral P., Morgan B.P., Rodriguez de Cordoba S. (2007). Gain-of-function mutations in complement factor B are associated with atypical hemolytic uremic syndrome. Proc. Natl. Acad. Sci. USA.

[97] Fremeaux-Bacchi V., Miller E.C., Liszewski M.K., Strain L., Blouin J., Brown A.L., Moghal N., Kaplan B.S., Weiss R.A., Lhotta K. (2008). Mutations in complement C3 predispose to development of atypical hemolytic uremic syndrome. Blood.

[98] Fang C.J., Fremeaux-Bacchi V., Liszewski M.K., Pianetti G., Noris M., Goodship T.H., Atkinson J.P. (2008). Membrane cofactor protein mutations in atypical hemolytic uremic syndrome (ahus), fatal Stx-HUS, C3 glomerulonephritis, and the HELLP syndrome. Blood.

[99] Tsai H.M. (2010). Pathophysiology of thrombotic thrombocytopenic purpura. Int. J. Hematol..

[100] Saland J.M., Ruggenenti P., Remuzzi G. (2009). Liver-kidney transplantation to cure atypical hemolytic uremic syndrome. J. Am. Soc. Nephrol..

[101] Motto D.G., Chauhan A.K., Zhu G., Homeister J., Lamb C.B., Desch K.C., Zhang W., Tsai H.M., Wagner D.D., Ginsburg D. (2005). Shiga toxin triggers thrombotic thrombocytopenic purpura in genetically susceptible ADAMTS13-deficient mice. J. Clin. Invest..

[102] Melton-Celsa A.R., O’Brien A.D. (2003). Animal models for STEC-mediated disease. Methods Mol. Med..

[103] Gunzer F., Hennig-Pauka I., Waldmann K.-H., Sandhoff R., Grone H.-J., Kreipe H.-H., Matussek A., Mengel M. (2002). Gnotobiotic piglets develop thrombotic microangiopathy after oral infection with enterohemorrhagic *Escherichia coli*. Am. J. Clin. Pathol..

[104] Myhal M.L., Laux D.C., Cohen P.S. (1982). Relative colonizing abilities of human fecal and K 12 strains of *Escherichia coli* in the large intestines of streptomycin-treated mice. Eur. J. Clin. Microbiol..

[105] Wadolkowski E.A., Burris J.A., O’Brien A.D. (1990). Mouse model for colonization and disease caused by enterohemorrhagic *Escherichia coli* O157:H7. Infect. Immun..

[106] Fujii J., Kita T., Yoshida S., Takeda T., Kobayashi H., Tanaka N., Ohsato K., Mizuguchi Y. (1994). Direct evidence of neuron impairment by oral infection with verotoxin-producing *Escherichia coli* O157:H- in mitomycin-treated mice. Infect. Immun..

[107] Isogai E., Isogai H., Kimura K., Hayashi S., Kubota T., Fujii N., Takeshi K. (1998). Role of tumor necrosis factor alpha in gnotobiotic mice infected with an *Escherichia coli* O157:H7 strain. Infect. Immun..

[108] Karpman D., Connell H., Svensson M., Scheutz F., Alm P., Svanborg C. (1997). The role of lipopolysaccharide and Shiga-like toxin in a mouse model of *Escherichia coli* O157:H7 infection. J. Infect. Dis..

[109] Brando R.J., Miliwebsky E., Bentancor L., Deza N., Baschkier A., Ramos M.V., Fernandez G.C., Meiss R., Rivas M., Palermo M.S. (2008). Renal damage and death in weaned mice after oral infection with Shiga toxin 2-producing *Escherichia coli* strains. Clin. Exp. Immunol..

[110] Kurioka T., Yunou Y., Kita E. (1998). Enhancement of susceptibility to Shiga toxin-producing *Escherichia coli* O157:H7 by protein calorie malnutrition in mice. Infect. Immun..

[111] Garcia A., Bosques C.J., Wishnok J.S., Feng Y., Karalius B.J., Butterton J.R., Schauer D.B., Rogers A.B., Fox J.G. (2006). Renal injury is a consistent finding in Dutch Belted rabbits experimentally infected with enterohemorrhagic *Escherichia coli*. J. Infect. Dis..

[112] Stearns-Kurosawa D.J., Collins V., Freeman S., Debord D., Nishikawa K., Oh S.Y., Leibowitz C.S., Kurosawa S. (2011). Rescue from lethal Shiga toxin 2-induced renal failure with a cell-permeable peptide. Pediatr. Nephrol..

[113] Sauter K.A., Melton-Celsa A.R., Larkin K., Troxell M.L., O’Brien A.D., Magun B.E. (2008). Mouse model of hemolytic-uremic syndrome caused by endotoxin-free Shiga toxin 2 (Stx2) and protection from lethal outcome by anti-stx2 antibody. Infect. Immun..

[114] Zotta E., Lago N., Ochoa F., Repetto H.A., Ibarra C. (2008). Development of an experimental hemolytic uremic syndrome in rats. Pediatr. Nephrol..

[115] Moon H.W., Whipp S.C., Argenzio R.A., Levine M.M., Giannella R.A. (1983). Attaching and effacing activities of rabbit and human enteropathogenic *Escherichia coli* in pig and rabbit intestines. Infect. Immun..

[116] Tzipori S., Wachsmuth I.K., Chapman C., Birden R., Brittingham J., Jackson C., Hogg J. (1986). The pathogenesis of hemorrhagic colitis caused by *Escherichia coli* O157:H7 in gnotobiotic piglets. J. Infect. Dis..

[117] Mohawk K.L., O’Brien A.D. (2011). Mouse models of *Escherichia coli* O157:H7 infection and shiga toxin injection. J. Biomed. Biotechnol..

[118] Al-Jumaili I., Burke D.A., Scotland S.M., al-Mardini H., Record C.O. (1992). A method of enhancing verocytotoxin production by *Escherichia coli*. FEMS Microbiol. Lett..

[119] MacLeod D.L., Gyles C.L. (1989). Effects of culture conditions on yield of Shiga-like toxin-II from *Escherichia coli*. Can. J. Microbiol..

[120] Lomas-Neira J., Perl M., Venet F., Chung C.S., Ayala A. (2012). The role and source of tumor necrosis factor-alpha in hemorrhage-induced priming for septic lung injury. Shock.

[121] Landoni V.I., de Campos-Nebel M., Schierloh P., Calatayud C., Fernandez G.C., Ramos M.V., Rearte B., Palermo M.S., Isturiz M.A. (2010). Shiga toxin 1-induced inflammatory response in lipopolysaccharide-sensitized astrocytes is mediated by endogenous tumor necrosis factor alpha. Infect. Immun..

[122] Landoni V.I., Schierloh P., de Campos Nebel M., Fernández G.C., Calatayud C., Lapponi M.J., Isturiz M.A. (2012). Shiga toxin 1 induces on lipopolysaccharide-treated astrocytes the release of tumor necrosis factor-alpha that alter brain-like endothelium integrity. PLoS Pathog..

[123] Barrett T.J., Potter M.E., Wachsmuth I.K. (1989). Bacterial endotoxin both enhances and inhibits the toxicity of Shiga-like toxin II in rabbits and mice. Infect. Immun..

[124] Taylor F.B., Toh C.H., Hoots W.K., Wada H., Levi M. (2001). Towards definition, clinical and laboratory criteria, and a scoring system for disseminated intravascular coagulation. On behalf of the scientific subcommittee on disseminated intravascular coagulation (DIC) of the International Society on Thrombosis and Haemostasis (ISTH). Thromb. Haemost..

[125] Stearns-Kurosawa D.J., Osuchowski M.F., Valentine C., Kurosawa S., Remick D.G. (2011). The pathogenesis of sepsis. Annu. Rev. Pathol..

[126] Remick D.G., Ward P.A. (2005). Evaluation of endotoxin models for the study of sepsis. Shock.

[127] Chart H., Smith H.R., Scotland S.M., Rowe B., Milford D.V., Taylor C.M. (1991). Serological identification of *Escherichia coli* O157:H7 infection in haemolytic uraemic syndrome. Lancet.

[128] Bitzan M., Moebius E., Ludwig K., Muller-Wiefel D.E., Heesemann J., Karch H. (1991). High incidence of serum antibodies to *Escherichia coli* O157 lipopolysaccharide in children with hemolytic-uremic syndrome. J. Pediatr..

[129] Chart H., Jenkins C. (1999). The serodiagnosis of infections caused by verocytotoxin-producing *Escherichia coli*. J. Appl. Microbiol..

[130] Ziegler E.J., Fisher C.J., Sprung C.L., Straube R.C., Sadoff J.C., Foulke G.E., Wortel C.H., Fink M.P., Dellinger R.P., Teng N.N. (1991). Treatment of gram-negative bacteremia and septic shock with HA-1a human monoclonal antibody against endotoxin. A randomized, double-blind, placebo-controlled trial. The HA-1a sepsis study group. N. Engl. J. Med..

[131] Angus D.C., Birmingham M.C., Balk R.A., Scannon P.J., Collins D., Kruse J.A., Graham D.R., Dedhia H.V., Homann S., MacIntyre N. (2000). E5 murine monoclonal antiendotoxin antibody in gram-negative sepsis: A randomized controlled trial. E5 study investigators. J. Am. Med. Asso..

[132] Deitch E.A. (2012). Gut-origin sepsis: Evolution of a concept. Surgeon.

[133] Matzinger P. (2002). The danger model: A renewed sense of self. Science.

[134] Wolfson J.J., May K.L., Thorpe C.M., Jandhyala D.M., Paton J.C., Paton A.W. (2008). Subtilase cytotoxin activates PERK, IRE1 and ATF6 endoplasmic reticulum stress-signalling pathways. Cell Microbiol..

